# Clinical risk factors and cancer risk of thyroid imaging reporting and data system category 4 A thyroid nodules

**DOI:** 10.1007/s00432-024-05847-7

**Published:** 2024-06-25

**Authors:** Jing Cheng, Bing Han, Yingchao Chen, Qin Li, Wenwen Xia, Ningjian Wang, Yingli Lu

**Affiliations:** 1 Department of Endocrinology and Metabolism, Shanghai Ninth People’s Hospital, School of Medicine, Shanghai Jiao Tong University, Shanghai, 200011 China; 2 Department of Pathology, Shanghai Ninth People’s Hospital, Shanghai Jiao Tong University School of Medicine, Shanghai, 200011 China

**Keywords:** Thyroid nodule, C-TIRADS, Age, FNA

## Abstract

**Purpose:**

Beyond the Thyroid Imaging Reporting and Data System (TIRADS) classification of thyroid nodules, additional factors must be weighed in the decision to perform fine needle aspiration (FNA). In this study, we aimed to identify risk factors for malignancy in patients with ultrasound-classified Chinese-TIRADS (C-TIRADS) 4 A nodules.

**Methods:**

Patients who underwent thyroid FNA at our institution between May 2021 and September 2022 were enrolled. We collected demographic data, including age, sex, previous radiation exposure, and family history. An in-person questionnaire was used to collect lifestyle data, such as smoking habits and alcohol consumption. Body mass index (BMI) was calculated. The serum levels of thyroid stimulating hormone (TSH), thyroid peroxidase antibody (TPOAb), and thyroglobulin antibody (TGAb) were measured. Prior to FNA, ultrasonic inspection reports were reviewed. The cytologic diagnoses for FNA of thyroid nodules followed the Bethesda System for Reporting Thyroid Cytopathology (2017).

**Results:**

Among the 252 C-TIRADS 4 A nodules, 103 were malignant. Compared to those in the benign group, the patients in the malignant group had a younger age (42.2 ± 13.6 vs. 51.5 ± 14.0 years, *P* < 0.001). Logistic regression showed that advanced age was associated with a lower risk of malignancy in C-TIRADS 4 A nodules (OR = 0.95, 95% CI 0.93 ~ 0.97, *P* < 0.001). We demonstrated a decreased risk of malignancy in patients with 48.5 years or older.

**Conclusion:**

Advanced age was associated with a decreased risk of malignancy in patients with C-TIRADS 4 A nodules. This study indicated that in addition to sonographic characteristics, patient age should be considered when assessing the risk of malignancy.

## Introduction

Thyroid nodules are common worldwide, with detection rates ranging between 19 and 67% using ultrasonography (Burman and Wartofsky 2015; Haugen et al. 2016). Most nodules are benign, but 7–15% are malignant, and most are papillary thyroid carcinomas (Haugen et al. 2016). The risk of thyroid cancer always guides the management strategies for patients with thyroid nodules. However, there is no perfect preoperative clinical, radiological, or molecular predictor of malignancy.

For thyroid nodules to be effectively managed, ultrasound is crucial for risk stratification (Tessler et al. 2017). Based on the ultrasound characteristics, the Thyroid Imaging Reporting and Data System (TIRADS) was developed to assess the risk of malignancy. According to the TIRADS, thyroid nodules are classified into different categories to determine their risk stratification. Different types of TIRADSs exist worldwide, including the American College of Radiology TIRADS, the Korean TIRADS, the European TIRADS, and the Chinese TIRADS (C-TIRADS) (Haugen et al. 2016; Russ et al. 2017; Zhou et al. 2020; Ha et al. 2021). Although the classification criteria for each version of the TIRADS are different, the management principles are similar; thyroid nodules with varying risks of malignancy should undergo fine needle aspiration (FNA) when their size reaches the corresponding threshold (Haugen et al. 2016; Russ et al. 2017; Tessler et al. 2017; Zhou et al. 2020; Ha et al. 2021). However, in addition to ultrasound characteristics and nodule size, many clinical factors, including age, sex, history of radiation exposure, and family history, are thought to predict nodule development or malignancy (Hegedüs 2004). According to previous studies, patient characteristics may influence the likelihood of a malignant thyroid nodule as assessed by FNA (Banks et al. 2008; Rago et al. 2010). In addition to a nodule’s TIRADS category, other factors must be considered when deciding whether to perform FNA.

In this study, we analysed the clinical risk factors for thyroid malignancies in patients with ultrasound-classified C-TIRADS 4 A nodules. We aimed to identify the important clinical predictors of malignancy in this subset of patients.

## Patients and methods

We reviewed a database of patients who underwent thyroid FNA between May 2021 and September 2022 at the Shanghai Ninth People’s Hospital Affiliated with the Shanghai Jiao Tong University School of Medicine, Department of Endocrinology. Before FNA, a recent thyroid ultrasound examination report was collected. The risk stratification of each thyroid nodule was determined according to the C-TIRADS by two experienced ultrasonographers. Patients who had ultrasound-confirmed C-TIRADS 4 A thyroid nodules and underwent FNA were enrolled. For patients with multiple nodules, only the nodule classified as C-TIRADS 4 A was subjected to FNA. The nodule size of each thyroid nodule was defined as the maximum diameter measured by ultrasonography. Patients who had undergone thyroidectomy or who were taking thyroid hormone preparations (including levothyroxine, triiodothyronine, or combinations of both) or antithyroid drugs (including methimazole or propylthiouracil) at the time of FNA evaluation were excluded.

Demographic data, including age, sex, history of radiation exposure, and family history of thyroid cancer, were collected. Weight and height were measured, and the body mass index (BMI) was calculated as weight in kilograms divided by height in meters squared. Lifestyle factor data, including smoking and drinking habits, were obtained using an in-person questionnaire. Smoking and drinking statuses were defined as currently smoking and drinking, respectively. The most recent serum thyrotropin (TSH), free thyrotropin (FT3), free tetraiodothyronine (FT4), thyroid peroxidase antibody (TPOAb), and anti-thyroglobulin (TGAb) levels were obtained before FNA. According to the manufacturer, a TPOAb concentration greater than 9.00 IU/ml was defined as serum TPOAb positivity. A TGAb concentration higher than 115 IU/ml was defined as serum TGAb positivity. The cytological diagnoses for FNA of the thyroid nodules followed the Bethesda System for Reporting Thyroid Cytopathology (2017) (Cibas and Ali 2017). According to this system, thyroid cytopathological reports are divided into six diagnostic categories: (i) nondiagnostic or unsatisfactory, (ii) benign, (iii) atypia of undetermined significance or follicular lesion of undetermined significance, (iv) follicular neoplasm or suspicious for a follicular neoplasm, (v) suspicious for malignancy, and (vi) malignant. Nodules were classified as malignant when the FNA cytology was “positive for malignant cells/malignant” or “suspicious for carcinoma” (including categories V and VI of the Bethesda system for cytopathology reporting).

The final diagnostic outcome based on FNA cytology was recorded as the presence or absence of malignancy. Age was analysed as a continuous and categorical variable as one of the primary clinical risk factors of interest. Age was analysed as a continuous variable to examine the effect of age on the likelihood of a malignant diagnosis. To further evaluate the malignancy rates in different groups, patients were divided into four age categories: (1) < 30 years, (2) 30–49 years, (3) 50–69 years, and (4) ≥ 70 years. Other clinical risk factors of interest, such as FT3, FT4, and TSH levels, nodule size, and BMI, were analysed as continuous variables. Sex, the presence of multiple nodules, TPOAb positivity, TGAb positivity, family history of thyroid cancer, and history of radiation exposure were analysed as categorical variables.

Data were analysed using SPSS version 26.0 (IBM Corporation, Armonk, NY, USA). Continuous variables are shown as the mean and standard deviation. Categorical variables are expressed as counts or proportions and analysed using the chi-square test. The risk factors and nodule malignancy risk were analysed using logistic regression. The results are expressed as odds ratios (ORs) and 95% confidence intervals (CIs). *P* values less than 0.05 were considered statistically significant. All reported *P* values were two-tailed.

## Results

### Baseline demographics

A total of 252 patients with a thyroid nodule identified on ultrasound as C-TIRADS 4 A were enrolled in our analysis. The patient demographic characteristics are summarised in Table 1. The mean age was 47.7 ± 14.6 years, and 205 patients were women. A total of 183 patients had multiple nodules, and the mean nodule size measured by ultrasound was 9.70 ± 7.76 mm. Four patients had a family history of thyroid cancer (1.6%), and two had a history of radiation exposure. The mean values of TSH, FT3, and FT4 were 2.46 ± 3.20 mIU/ml, 3.35 ± 0.51 pg/ml, and 0.98 ± 0.96 ng/dl, respectively. The percentages of TGAb and TPOAb positivity were 20.6% and 23.40%, respectively. Sixteen patients were smokers, and 17 were regular alcohol drinkers **(**Table 1**)**.

**Table 1:** Patient Demographics with C-TIRADS 4 A Thyroid Nodules

Variables	
No of patients	252
Age (mean ± SD, years)	47.7 ± 14.6
Female n (%)	205 (81.30%)
Nodule size (mean ± SD, mm)	9.70 ± 7.76
Multiple nodules n (%)	183(72.60%)
Family history of thyroid cancers n (%)	4 (1.60%)
History of radiation exposure n (%)	2 (0.80%)
TSH (mean ± SD, µIU/mL)	2.46 ± 3.20
BMI (mean ± SD, kg/m^2)	23.87 ± 3.75
FT3 (mean ± SD, pg/ml)	3.35 ± 0.51
FT4 (mean ± SD, ng/dL)	0.98 ± 0.96
TGAb positivity n (%)	52 (20.60%)
TPOAb positivity n (%)	59 (23.40%)
Smoking n (%)	16 (6.30%)
Drinking n (%)	17 (6.70%)

*TSH*, serum thyrotropin (µIU/mL); *BMI*, body mass index (kg/m2); *FT3*, free triiodothyronine (pg/ml); *FT4*, thyroxin (ng/dL); TGAb, thyroglobulin antibody; TPOAb, thyroid peroxidase antibody; SD, standard deviation.

Among the 252 patients whose thyroid nodules were determined to be C-TIRADS 4 A, 103 had malignant nodules, all of which were papillary carcinomas. By comparing the clinical variables between the benign and malignant groups, we found that the mean age was lower in the malignant group (42.2 ± 13.6 vs. 51.5 ± 14.0 years, *P* < 0.001). However, no significant differences were observed in the percentage of women, number of patients with multiple nodules, nodule size, history of radiation exposure, or family history of thyroid cancer. Additionally, no differences were detected in BMI, TSH, FT3, FT4, percentage of TGAb positivity, or TPOAb positivity among the patients **(**Table 2**)**.

**Table 2:** Clinical Characteristics of Patients with Benign and Malignant C-TIRADS 4 A Nodules

Variables	Group		
	Benign group	Malignant group	*P* value
Number of patients	149	103	
Female (%)	82.60%	79.60%	0.56
Mean age (years)	51.5 ± 14.0	42.2 ± 13.6	< 0.001
Mean nodule size by ultrasound (mm)	10.3 ± 8.6	8.9 ± 6.3	0.06
Multiple nodules (%)	75.80%	68%	0.17
Family history of thyroid cancers (%)	1.3%(2/149)	1.9%(2/103)	0.71
History of radiation exposure (%)	0(0/149)	1.9%(2/103)	0.09
TSH (µIU/mL)	2.24 ± 2.61	2.77 ± 3.89	0.19
BMI (kg/m^2)	24.12 ± 3.90	23.52 ± 3.50	0.21
FT3 (pg/ml)	3.35 ± 0.57	3.33 ± 0.42	0.76
FT4 (ng/dL)	1.04 ± 1.24	0.89 ± 0.16	0.24
TGAb positivity (%)	20.80%	18.40%	0.67
TPOAb positivity (%)	25.50%	17.50%	0.17

*TSH*, serum thyrotropin (µIU/mL); *BMI*, body mass index (kg/m2); *FT3*, free triiodothyronine (pg/ml); *FT4*, thyroxin (ng/dL); TGAb, thyroglobulin antibody; TPOAb, thyroid peroxidase antibody.

### Association between risk factors and malignancy in C-TIRADS 4 a nodules

**Fig. 1:**
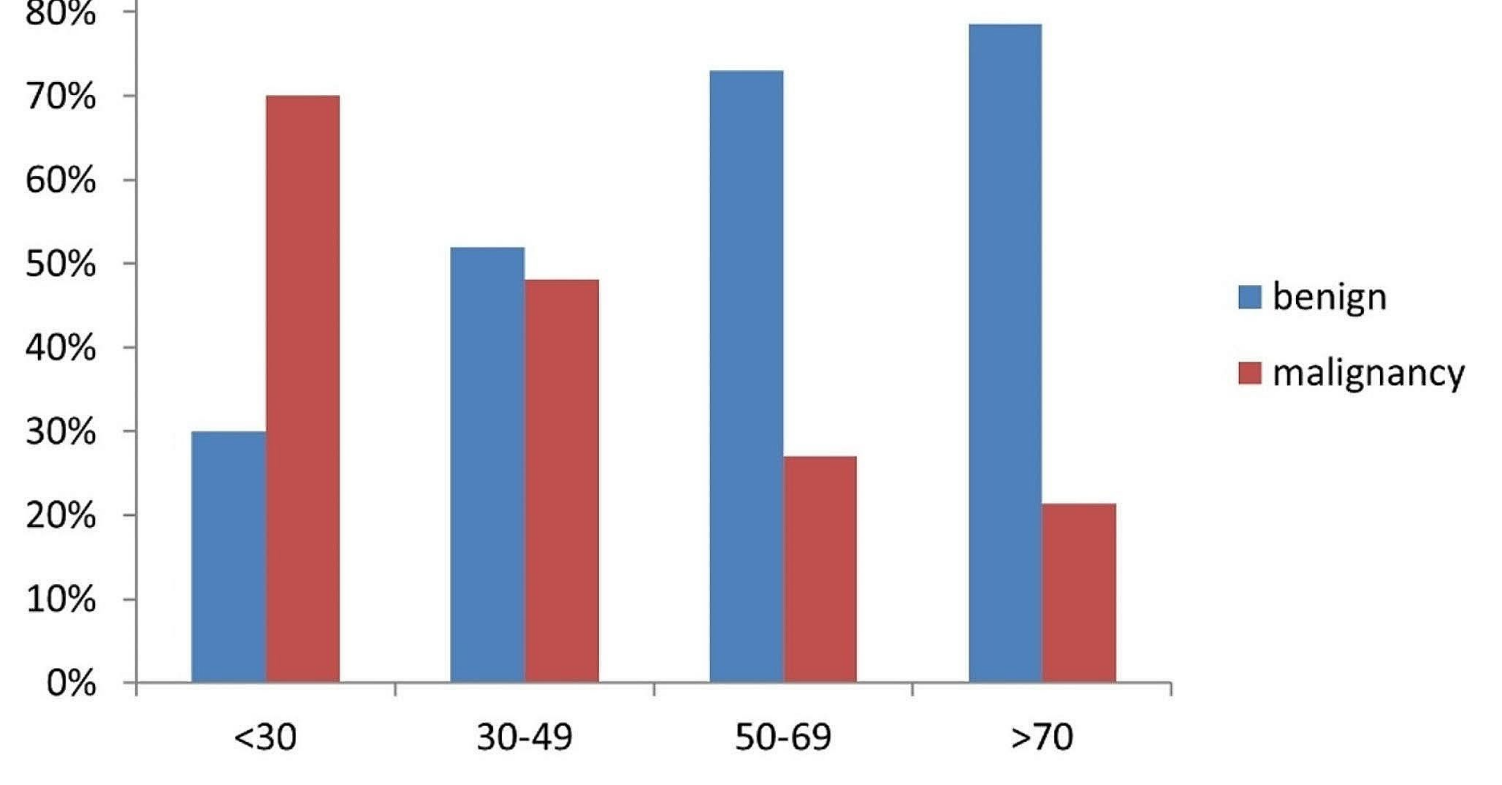
Frequencies of Benign and Malignant C-TIRADS 4 A Nodules among different age groups

Logistic regression analysis revealed that advanced age was significantly associated with a reduced risk of malignancy in C-TIRADS 4 A nodules (OR = 0.95, CI 0.93–0.97, *P* < 0.001) (Table 3). According to age group, we found that the rate of malignancy decreased with advancing age (70% for those aged < 30 years; 48.1% for those aged 30–49 years; 27% for those aged 50–69 years; and 21.4% for those aged ≥ 70 years) **(**Fig. 1**).**

**Table 3:** Association between the Risk Factors and Malignancy Risk in Patients with C-TIRADS 4 A Nodules

Variables	OR	CI (5-95%)	*P* value
Female	0.92	0.45–1.87	0.82 < 0.001 - 0.04 < 0.001 0.008 0.16 0.56 0.36 0.72 0.96 1
Age	0.95	0.93–0.97	
< 30	Ref	-	
30–49	0.38	0.15–0.94	
50–69	0.16	0.06–0.43	
≥ 70	0.12	0.02–0.57	
Nodule size by ultrasound	0.97	0.94–1.01	
Multiple nodules (%)	1.22	0.64–2.32	
TSH	1.04	0.95–1.14	
BMI	0.99	0.91–1.07	
Family history of thyroid cancer	0.95	0.12–7.63	
History of radiation exposure	0	0	

*OR*, odds ratio; *CI*, confidence interval; Ref, reference value; TSH, serum thyrotropin (µIU/mL); BMI, body mass index (kg/m2).

### Cut-off value of age for predicting malignancy risk

**Fig. 2:**
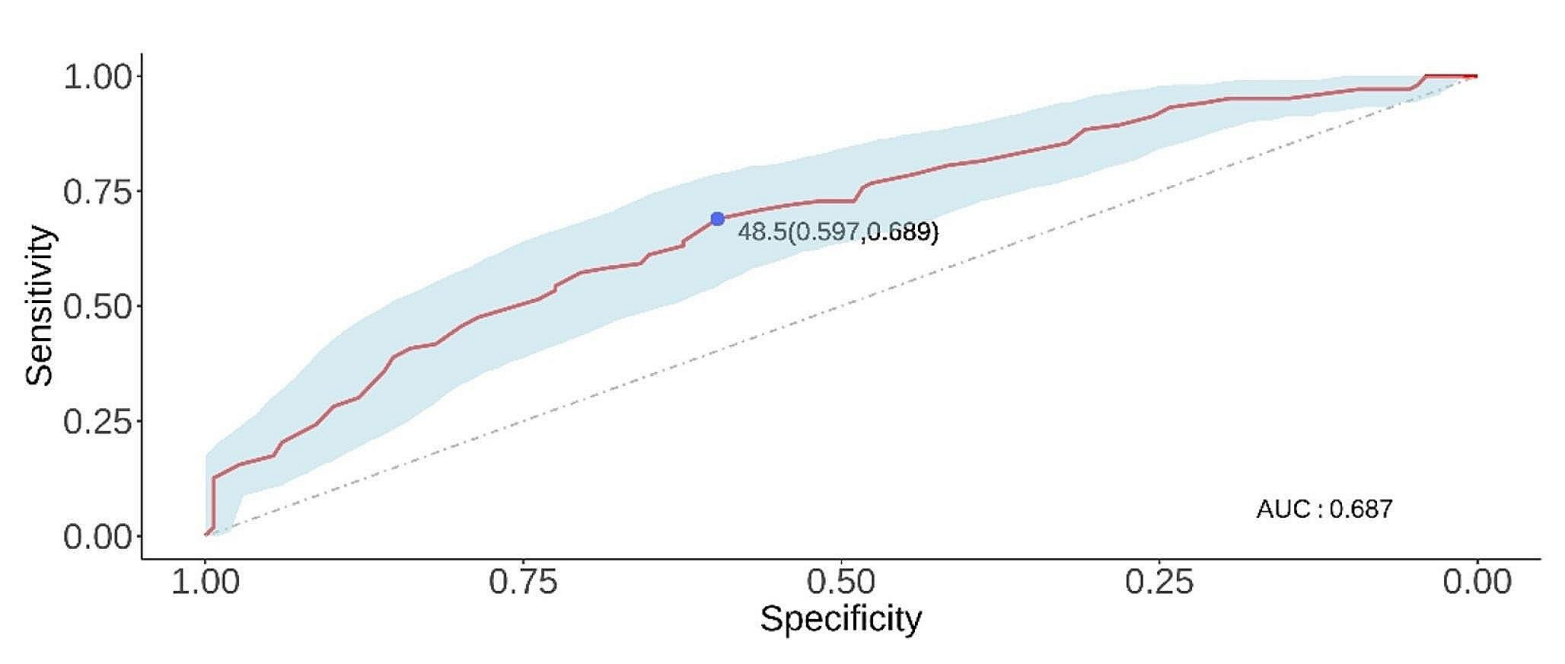
Optimal cut-off value for age derived from the ROC curve

To determine the optimal age cut-off value that balances sensitivity and specificity, thus effectively predicting the risk of malignancy, we performed an analysis utilising a receiver operating characteristic (ROC) curve **(**Fig. 2**)**. With age as a categorical variable, a cut-off of 48.5 years was identified, which allowed us to demonstrate a decreased risk of malignancy in patients aged 48.5 years or older (area under the curve = 0.687, *P* < 0.001).

## Discussion

Ultrasound is crucial in determining the risk stratification for effectively managing thyroid nodules (Tessler et al. 2017). Using ultrasound, thyroid nodules can be assessed for malignancy risk based on morphological characteristics. Ultrasound can also reveal the spatial location of nodules within the thyroid gland, the relationship between nodules and the thyroid capsule, and the relationship between nodules, nearby blood vessels, and nerves. Furthermore, preoperative ultrasound can optimise surgical treatment, change surgical procedures, facilitate the complete removal of lesions, and reduce the risk of recurrence. Follow-up ultrasound is also important for patients with postoperative thyroid cancer (Kouvaraki et al. 2003; Mazzaglia 2010; Tessler et al. 2017). Although the guidelines recommend that patients with thyroid nodules with suspicious ultrasound signs should undergo FNA to detect malignant nodules, many thyroid surgeries in China are based only on ultrasound findings or other relevant clinical evidence instead of FNA results, resulting in overtreatment and irreparable harm to patients (Zhou et al. 2020). The patient characteristics and rate of thyroid cancer on ultrasound should be considered to assess a patient’s cancer risk prior to FNA. We aimed to determine patient characteristics that influence cancer risk using the same ultrasound classification category. Age was significantly associated with the risk of malignancy in patients with C-TIRADS 4 A nodules. The rate of thyroid nodule malignancy on FNA was higher in younger patients than in older patients.

Although epidemiological analyses have demonstrated a positive association between thyroid nodule formation and age, the relationship between age and thyroid cancer risk remains controversial (Reiners et al. 2004; Kwong et al. 2015; Gong et al. 2022). A study by Laurel et al. reviewed 3,981 consecutive patients who underwent thyroid FNA between February 2002 and December 2009 at the University of Wisconsin. According to FNA findings, the risk of thyroid nodule malignancy varied depending on the patient’s age. The incidence of thyroid cancer diagnosed using FNA was higher in younger patients (aged ≤ 45 years) (Bessey et al. 2013). Kwong et al. also demonstrated that the incidence of clinically relevant thyroid nodules increased with advancing age, whereas the risk of malignant nodules decreased (Kwong et al. 2015). However, none of the studies included the ultrasound features of the thyroid nodules or the specific reason why FNA was performed on each patient. Our study analysed the clinical characteristics of patients with thyroid nodules with the same ultrasound classification category (C-TIRADS 4 A), which was meaningful. When thyroid nodules are larger than 15 mm, Chinese guidelines recommend FNA to determine whether they are malignant (Zhou et al. 2020). Our study indicated that age was significantly associated with malignancy using FNA in this subgroup. In addition to the ultrasound thyroid nodule category, age should also be considered to determine whether the clinician should proceed with further confirmatory tests or periodic follow-up.

Female sex has also been recognised as a risk factor for papillary thyroid cancer. The incidence of thyroid cancer is three- to fourfold greater in women than in men (Rahbari et al. 2010). However, other studies have indicated that men are more likely than women to have a malignant thyroid nodule (Bessey et al. 2013; Nguyen et al. 2023). Many studies have demonstrated that the incidence of differentiated thyroid cancer is similar between men and women and in children and adults (Farahati et al. 1997; Sassolas et al. 2009; Colonna et al. 2020; Vaccarella et al. 2021). In this study, although most patients were women, we did not find a significant association between sex and cancer risk in the C-TIRADS 4 A thyroid nodules.

Nodule size is an important prognostic factor for patients with thyroid cancer. Moreover, it is important to determine whether a C-TIRADS 4 A thyroid nodule requires further FNA or surgery according to the guidelines (Haugen et al. 2016; Russ et al. 2017; Tessler et al. 2017; Zhou et al. 2020; Ha et al. 2021). Interestingly, this study did not find an association between thyroid nodule size and cancer risk among C-TIRADS 4 A thyroid nodules. Nodule size alone is not a reliable indicator of thyroid cancer (Frates et al. 2006). No consensus exists as to whether nodule size can accurately predict thyroid malignancies. Some studies have suggested that a thyroid nodule ≥ 2 cm was associated with an increased risk of well-differentiated thyroid cancer (Kamran et al. 2013). Other studies have demonstrated that a nodule ≥ 3.0–4.0 cm was associated with an increased probability of malignancy (McCoy et al. 2007; Koo et al. 2016; Jinih et al. 2020; Boonrod et al. 2021). However, other groups have reported that a smaller nodule size is associated with a greater malignancy rate (Castro et al. 2011). One of the primary limitations of these studies is that the final histopathological diagnoses were available only for nodules that underwent surgical resection. Considering that many patients opt for active surveillance or minimally invasive interventions rather than traditional surgery, these studies could have been subject to selection bias. This study analysed the relationship between nodule size and malignancy using FNA and did not find an association between nodule size and the risk of thyroid cancer. This is consistent with previous reports that thyroid nodule size does not accurately predict the risk of thyroid malignancy, irrespective of FNA cytology (Cavallo et al. 2017).

Some studies have demonstrated that increased serum TSH levels increase the risk of thyroid cancer, particularly papillary thyroid cancer (Boelaert et al. 2006; Fiore et al. 2009; Rago et al. 2010; Fiore and Vitti 2012). However, other studies have reported an inverse association between TSH levels and thyroid cancer (Biondi et al. 2005; Gudmundsson et al. 2012; Rinaldi et al. 2014; Yuan et al. 2020). A Mendelian randomisation study indicated a protective role of high TSH levels in thyroid cancer (Yuan et al. 2020). Moreover, a case-control study showed that low TSH levels might predispose patients to thyroid cancer (Rinaldi et al. 2014). Our study revealed no association between TSH levels and thyroid cancer, which is consistent with the findings of a nationwide cohort study of 63,143 patients with hypothyroidism compared with the general population (Kitahara et al. 2018).

This study has several limitations. First, the average nodule size on ultrasound was 9.70 ± 7.76 mm, which was much less than the 15 mm recommended by the guidelines. Many patients chose to undergo FNA rather than follow-up. This indicated that a high ultrasound category (category 4 A and above) caused anxiety in patients, making them more likely to receive further testing, even if the nodule size was less than the size recommended by the guidelines. Second, there was no association between cancer risk and certain known risk factors, such as a history of radiation exposure, a history of preexisting thyroid disease, or a family history of thyroid cancer, which might be because of the small sample size. Most patients were sporadic; only four patients had a family history of thyroid cancer (1.6%), and two had a history of radiation exposure. Additionally, 103 of the 252 nodules were diagnosed as malignant or suspicious of carcinoma. This percentage exceeded the recommended risk for C-TIRADS category 4 A nodules. This could be attributed to the ultrasound and cytological diagnoses. The precision of diagnostic ultrasonography relies considerably on the skill of the operator. Even when a single risk stratification system (C-TIRADS) is used, the inter-observer variability for thyroid ultrasonography can be high (Hoang et al. 2018). It is possible that the ultrasonographers underestimated the malignancy risks of some thyroid nodules. Furthermore, the outcome of this study was cytological findings, which might vary from the histological results. Regrettably, definitive histopathological diagnoses for the thyroid nodules were unattainable, given that most patients did not receive surgical treatment at our facility. Nevertheless, our study was meaningful, as it revealed that the cancer risk ascertained by FNA within identical ultrasound categories varied with age. This insight is crucial for patient counselling and for refining the application and analysis of FNA outcomes.

## Conclusion

We demonstrated that age was significantly associated with the cancer risk of thyroid nodules classified as category C TIRADS 4 A on ultrasound. The rate of thyroid nodule malignancy on FNA was higher in younger patients than in older patients. These findings indicate that in addition to sonographic characteristics, patient age should be considered when assessing the risk of malignancy in thyroid nodules.

## Data Availability

No datasets were generated or analysed during the current study.
